# Modeling the Dynamic Recrystallization and Flow Curves Using the Kinetics of Static Recrystallization

**DOI:** 10.3390/ma12183024

**Published:** 2019-09-18

**Authors:** Valeriy Shkatov, Igor Mazur

**Affiliations:** Institute of metallurgy, Lipetsk State Technical University, 398055 Lipetsk, Russia

**Keywords:** dynamic recrystallization, static recrystallization, hot deformation, flow stress, critical strain, modeling

## Abstract

The results of modeling the dynamic recrystallization of steels during hot deformation on the basis of information on their static recrystallization kinetics are presented. The results of predicting the amount of deformation accumulated in the metal under the conditions of dynamic recrystallization development were used for calculating the metal flow curves. The model was validated by comparing the calculated flow curves with the experimental flow curves determined on the 1045 steel by means of hot torsion tests carried out from 1000 °C to 1100 °C and at strain rates from 0.1 to 10 s^‒1^. The difference between the experimental and predicted flow stress values did not exceed 6%. The influence of the chemical element content in low-alloyed steels on the magnitude of the critical strain for the initiation of dynamic recrystallization is assessed. The method of predicting the kinetics of dynamic recrystallization by recalculating the kinetics of static recrystallization to the conditions of continuous growth of the strain degree during metal deformation implemented in the model can be used in designing and optimizing technologies associated with metal hot forming processes.

## 1. Introduction

The design and optimization of technologies which use models of dynamic recrystallization (DRX) and static recrystallization (SRX) during hot plastic deformation of structural steels are among the promising ways of obtaining high values of strength and plastic properties of the finished metal products. The conditions and degree of the recrystallization process development also affect the level of metal flow stress during deformation, the accuracy of which depends on the accuracy of calculating the energy parameters in metal hot forming processes [1,2,3].

Static recrystallization is a process of the formation and growth of new grains in a deformed matrix, the grains being free from distortions or much more perfect than this matrix and separated from it by high grain boundary angles [4]. There is a fairly large number of studies on the effect of hot deformation parameters on static recrystallization of steels and the development of mathematical models of static recrystallization for individual steel grades or groups of steel with a similar composition [5,6,7,8,9,10,11]. In [12,13], mathematical models of the kinetics of static recrystallization of carbon and low-alloyed steels after hot deformation are proposed, allowing us to take into account the content of chemical elements in steel, along with the deformation parameters.

With dynamic recrystallization, which develops directly during hot plastic deformation, the softening processes proceed simultaneously with the hardening processes. In the grains formed during dynamic recrystallization, due to the continuing deformation, the dislocation density gradually increases, which creates conditions for the nucleation and growth of new recrystallized grains [4]. Currently, a large number of mathematical models of dynamic recrystallization during hot deformation have been proposed based on different principles and approaches. The reviews [14,15,16] provide a comparative description of various types of these models: Phenomenological, those based on the theory of defects in the crystal structure of metals, those using cellular automata, the Monte–Carlo method, etc. Their common drawback is the inability to predict the effect of the chemical composition of steel on recrystallization processes, e.g., when the content of alloying elements changes within the grade composition of steel or when the steel grade changes. It is required that the constants of these models be determined by the results of the experiment for each steel grade (or a group of steel grades similar in composition) [17,18,19], which significantly limits the scope of the models.

A common feature of both static and dynamic recrystallization is the movement of high angle grain boundaries. The driving force for both types of recrystallization is the excess bulk energy accumulated during plastic deformation [4]. Thus, the mechanism of static and dynamic recrystallization is the same; the difference lies in the features of their implementation: After the deformation is completed or during deformation under conditions of continuous growth of the strain degree. It should be expected that the differences in the static and dynamic recrystallization kinetics are also due to the conditions of implementing these processes and, hence, can be estimated by calculation. The possibility of predicting dynamic recrystallization based on the information on the static recrystallization kinetics was first demonstrated in [20,21] using the example of predicting critical strain for dynamic recrystallization and strain to the peak stress during dynamic recrystallization of steels using the equations of static recrystallization kinetics.

This article presents a mathematical model for predicting dynamic recrystallization during hot deformation of carbon and low alloyed steels, which is based on the method of recalculating the kinetics of static recrystallization to the conditions of continuous growth of the degree of strain during metal deformation. The results of the calculation of the dynamic recrystallization kinetics were used to predict the flow stress of steel and the critical strain for the onset of dynamic recrystallization.

## 2. Material and Experiments

The experimental study of the effect of hot deformation parameters on the flow stress of metal was carried out on the 1045 steel containing 0.43% C, 0.64% Mn, 0.21% Si, 0.025% P, 0.028% S (values are in wt.%). The 1045 steel is produced in large volumes and is widely used in engineering. This steel is supplied in the form of hot-rolled sheets, bars, and special-shaped products.

The torsion test was performed on the STD 812 torsion plastometer. The torsion specimens with a gauge length (L) of 8 mm and 3 mm in radius (R) were heated inductively in a vacuum working chamber at a speed of 10 °C/s to a temperature of 1150 °C and allowed to remain for 600 s. The samples were then cooled at a rate of 5 °C/s to a deformation temperature of 1000, 1050, and 1100 °C, and, after a 30 s pause to stabilize the temperature, were subjected to strain at a strain rate of 0.1, 1, and 10 s^−1^ until the strain reached 4.0.

The measured torque *Γ* and number of revolutions *N* were converted to von Mises effective stress (σ) and strain (ε) using the following equations [22]:

(1) $$\sigma =\frac{3.3\sqrt{3\;}\Gamma }{2\pi R^{3}},$$

(2) $$\varepsilon =\frac{2\pi RN}{\sqrt{3}L}$$

In order to determine the grain size of austenite before deformation, the samples were heated in a DIL805A/D quenching-deformation dilatometer in a mode simulating the heating of samples before deformation and then subjected to accelerated cooling with a stream of argon. The austenite grain in quenched samples was detected by etching in a 5% alcoholic solution of picric acid. The grain size was measured on an optical microscope at magnification ×500 using the chord method.

The critical strain for the dynamic recrystallization $\varepsilon_{с}$ was determined, according to the method proposed by the authors [23,24], as a strain corresponding to the inflection point on the strain hardening rate *θ*$\;=\frac{\partial \sigma }{\partial \varepsilon }$ versus the flow stress *σ* curve.

## 3. Formulation of the Model

### 3.1. Modeling of Strain Hardening

**Figure 1:**
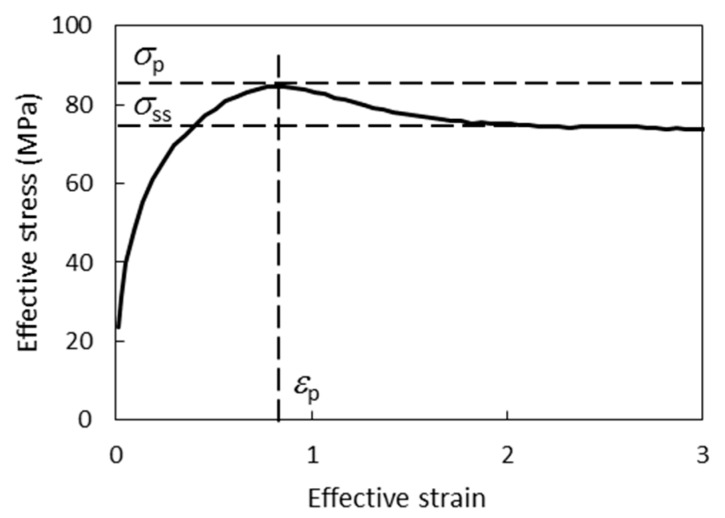
Hot torsion flow curve determined at 1000 °C and strain rate 1 s^−1^.

The torsion experiments on 1045 steel samples resulted in flow curves that carry information on the metal strain resistance level, the presence and intensity of the softening processes under given conditions. Flow curves are typical for the case when metal softening during hot deformation is caused by conventional dynamic recrystallization (Figure 1). As the strain increases, the flow stress *σ* increases to a certain peak value *σ*_p_ of the corresponding peak strain *ε*_p_. Then *σ* decreases smoothly until the value corresponding to the steady flow stress *σ_ss_*, at which equilibrium is reached in the processes of strain hardening and softening.

After reaching the critical strain for the onset of dynamic recrystallization *ε*_c_, the processes of metal hardening proceed simultaneously with the softening processes. The solution to the problem of predicting the flow stress in these conditions involves the development of mathematical models of each of these processes.

In order to develop a strain hardening model for each experimental flow curve, the critical strain for dynamic recrystallization was determined. It was established that at a strain rate of $\dot{\varepsilon }$ = 0.1 s^−^^1^, an increase in temperature from 1000 to 1100 °C leads to a decrease in $\varepsilon_{c}$ from 0.199 to 0.158, and at $\dot{\varepsilon }$ = 1 s^−^^1^, from 0.288 to 0.230. For all flow curves with $\dot{\varepsilon }$ ≤ 1 s^−1^, the ratio *ε*_c_/*ε*_p_ practically did not change and was in the range of 0.37–0.39.

Experimental flow curves determined at a strain rate of 10 s^−1^ are characterized by significant fluctuations in the readings of the torque sensor. In this connection, the critical strain at a strain rate of 10 s^−1^ was estimated as $\varepsilon_{c}$ = 0.38$\varepsilon_{p}$.

To obtain the equation of the strain hardening curve, the experimentally obtained flow curves at temperatures of 1000, 1050, and 1100 °C; and strain rates of 0.1, 1, and 10 s^−1^ were used. In order to eliminate the effect of dynamic recrystallization on the flow stress on each stress-strain curve, 10 values of flow stress were measured at a strain below $\varepsilon_{c}$. According to the results of measurements, an array of data was generated containing 90 values of flow stress for the corresponding strain values, strain rate, and strain temperature.

From the array of experimental data via the least squares method using linearization transformations of the variables, the equation of the relation between the flow stress before the onset of dynamic recrystallization, and deformation parameters was obtained:

(3) $$\sigma =1.498\;\varepsilon^{0.271}{\dot{\varepsilon }}^{0.129}\exp\left(\frac{5317.0}{T}\right),$$

where ε is the strain, $\dot{\varepsilon }$ is the strain rate, с^−1^, and T is the temperature, *К*.

**Figure 2:**
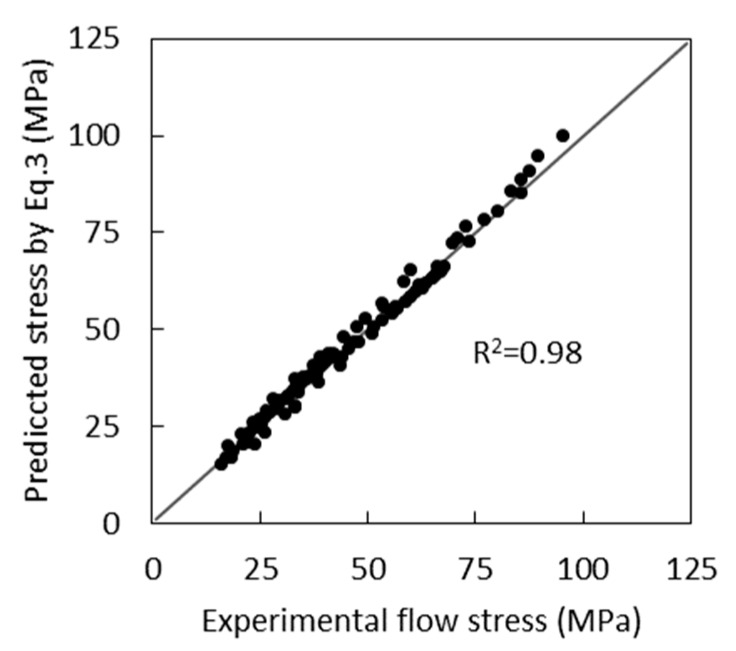
Comparison between experimental flow stress and predicted values by Equation (3).

**Figure 3:**
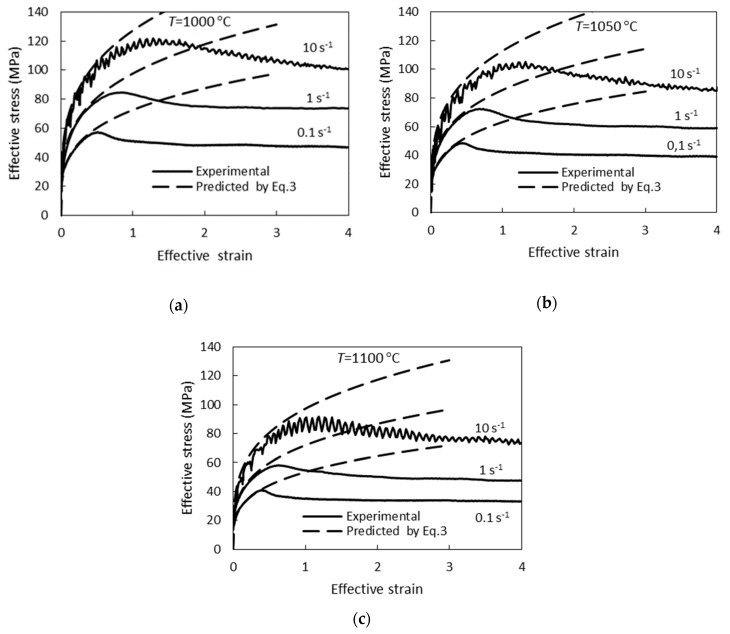
Experimental hot torsion flow curve determined at (**a**) 1000 °C, (**b**) 1050 °C, and (**c**) 1100 °C and curve predicted by Equation (3).

The mean absolute error in the prediction of the flow stress by Equation (3) is 1.8 MPa. The results of the calculation of *σ* by Equation (3) are compared with the experimental data in Figure 2, and the experimentally determined flow curves along with the curves calculated by Equation (3) are shown in Figure 3.

### 3.2. Static Recrystallization

The kinetics of static recrystallization was described by a modified Johnson–Mehl–Avrami–Kolmogorov (JMAK) equation [25]:

(4) $$X^{SRX}=1-\exp\left[-B{\left(\frac{t}{t_{0.5}}\right)}^{n}\right],$$

where $X^{SRX}$ is the degree of recrystallization, fraction, $t_{0.5}$ is the recrystallization time by 50%, s, t is the current time, s, B=−ln 0.5, and n is the coefficient.

The value of $t_{0.5}$ was determined from the mathematical description of the static recrystallization kinetics of hot-deformed austenite in C–Mn/Si steels, C–Mo steels, and Nb/V micro-alloyed steels obtained in [13], following the hot torsion tests:

(5) $$t_{0.5}=3.754\cdot 10^{-4}\exp\left(-7.869\cdot 10^{-5}Q\right)\varepsilon^{-4.3d_{0}^{-0.169}}{\dot{\varepsilon }}^{-0.53}d_{0}^{1.09}\exp\left(\frac{Q}{RT}\right),$$

where ε is the strain, $\dot{\varepsilon }$ is the strain rate, s^−1^, $d_{0}$ is the austenite grain size before deformation, μm, T is the temperature, K, R is the universal gas constant, J/(mol·K).

In Equation (4), the activation energy Q is a function of the chemical composition of the steel and is calculated as:

(6) $$Q=148636.8-71981.3\left[C\right]+56537.6\left[\mathrm{Si}\right]+21180\left[\mathrm{Mn}\right]+121243.3\left[\mathrm{Mo}\right]++64469.6\left[V\right]+109731.9{\left[\mathrm{Nb}\right]}^{0.15}.$$

The average grain size of austenite after the completion of static recrystallization in C–Mn/Si low-alloyed steels was found by the formula [26]:

(7) $$d^{SRX}=343d_{0}^{0.4}\varepsilon^{0.5}\exp\left(-\frac{45000}{RT}\right),$$

and for Nb/V micro-alloyed steels, the dependence [27] was used:

(8) $$d^{SRX}=1.1d_{0}^{0.67}\varepsilon^{-0.67}.$$

To determine the average grain size of austenite in a partially recrystallized structure, we used the equation [28]:

(9) $$d={\left(X^{SRX}\right)}^{4/3}d^{SRX}+{\left(1-X^{SRX}\right)}^{2}d_{0}.$$

### 3.3. Dynamic Recrystallization and Flow Stress

The algorithm for predicting dynamic recrystallization by recalculating the kinetics of static recrystallization to the conditions of continuous growth of the degree of strain during metal deformation is based on the rule of additivity. The rule of additivity was introduced by Avrami [29] and allows one to divide, for computational purposes, the reaction into isothermal (or isostrain in the present case) segments. The volume fractions predicted to form in each segment can be summed to give the total expected transformed volume fraction. It only holds if the reaction is isokinetic, but has been used effectively in predicting non isothermal recrystallization of ferrite [30], non-isothermal austenite-ferrite, and austenite-pearlite transformation in steels [31], etc.

The strain increase curve was represented as a combination of segments with a fixed strain of small magnitude. Here it was assumed that for the *i*-th segment of strain equals $\varepsilon_{i}$ and is kept constant for the time $\Delta t_{i}$. For the (*i*+1)-th segment, strain increases instantly to $\varepsilon_{i+1}$ and is kept constant for time $\Delta t_{i+1}=\Delta t_{i}=\Delta t$.

The degree of dynamic recrystallization during the first strain segment (i.e., at strain $\varepsilon_{1}$ for the time $t_{1}=\Delta t)$ was calculated:

(10) $$X_{1}^{DRX}=1-\exp\left[-0.693{\left(\frac{\Delta t}{t_{0.5}\left(\varepsilon_{1}\right)}\right)}^{n}\right].$$

During the second strain segment, recrystallization will continue to develop as if the degree of recrystallization of $X_{1}^{DRX}$ was obtained at strain $\varepsilon_{2}$ by the time of the beginning of the second strain segment. The time required to obtain the degree of recrystallization $X_{1}^{DRX}$ at a strain $\varepsilon_{2}$ was calculated by the formula:

(11) $$t_{2}^{*}=t_{0.5}\left(\varepsilon_{2}\right){\left[\frac{\ln\left(1/\left(1-X_{1}^{DRX}\right)\right)}{0.693}\right]}^{\frac{1}{n}}.$$

The obtained time $t_{2}^{*}$ was summed up with the increment Δt, and the degree of dynamic recrystallization at a strain $\varepsilon_{2}$ was calculated (during the second segment of strain):

(12) $$X_{2}^{DRX}=1-\exp\left[-0.693{\left(\frac{t_{2}^{*}+\Delta t}{t_{0.5}\left(\varepsilon_{2}\right)}\right)}^{n}\right]$$

The calculation procedure is repeated for the following strain segments.

This algorithm makes it possible to calculate the kinetics of dynamic recrystallization only at the initial stage of the process and can be used to predict critical strain for the onset of dynamic recrystallization.

With the further development of dynamic recrystallization in the resulting recrystallized grains, the dislocation density gradually increases due to continuing strain, which creates conditions for the nucleation and growth of new recrystallized grains, resulting in simultaneously running of multiple recrystallization cycles developing in a deformable metal. To account for this effect, the following procedure was used. When calculating the first cycle of dynamic recrystallization, the resulting recrystallized metal fraction was successively divided into k volumes of small size $V_{j}$ ($\sum_{j=1}^{k}V_{j}=1$). In each recrystallized volume $V_{j}$ from the moment of its formation (the completion of recrystallization in this volume), the kinetics of dynamic recrystallization was calculated according to the same scheme as for the first recrystallization cycle (Equations (4)–(12)), but under new initial conditions: Strain $\varepsilon_{ij}=0$; the grain size before deformation $d_{j}^{\left(0\right)}$ is equal to the size of the recrystallized grain in the volume. The strain accumulated in the volume $V_{j}$ at the *i*-th point of time at the degree of recrystallization of this volume $X_{ij}^{DRX}\;$ was calculated as:

(13) $$\varepsilon_{ij}^{a}=\varepsilon_{ij}\left(1-X_{ij}^{DRX}\right).$$

As a result, after the completion of the first recrystallization cycle, the recrystallization kinetics was calculated simultaneously for k metal volumes, each of which was characterized by its initial conditions. The strain accumulated in the metal at the *i*-th moment of time was calculated by the formula:

(14) $$\varepsilon_{i}^{a}=\overset{k}{\underset{j=1}{\sum }}\varepsilon_{ij}^{a}V_{j}.$$

The equation of strain hardening of steel during hot deformation (3) allows us to predict the flow stress in the absence of dynamic recrystallization. If dynamic recrystallization develops during the deformation, the value of the flow stress in this case is determined not by the strain $\varepsilon_{i}$, but by the amount of strain accumulated in the metal $\varepsilon_{i}^{a}$, depending on the degree of dynamic recrystallization. Therefore, to calculate the flow curves $\sigma^{DRX}\left(\varepsilon \right)$ during dynamic recrystallization in Equation (3), the strain *ε* was replaced by the accumulated strain $\varepsilon^{a}$

(15) $$\sigma^{DRX}\left(\varepsilon \right)=\;\sigma \left(\varepsilon^{a}\right).$$

## 4. Results and Discussion

The algorithms for predicting the kinetics of dynamic recrystallization and the flow stress of steel were programmed using OBJECT PASCAL language in the DELPHI application development environment. The program is designed to predict the kinetics of dynamic recrystallization, and on this basis calculate the metal flow stress. The program is used for hot deformation of carbon and low-alloyed steels, including those micro-alloyed with niobium and vanadium. The input information concerns the content of chemical elements in steel, the austenite grain size before deformation, strain value, strain rate, and temperature. The program calculates the critical strain for dynamic recrystallization, the degree of dynamic recrystallization, the austenite grain size and flow stress for a given steel chemical composition, and deformation parameters, also making it possible to obtain graphic displays of metal flow curves and austenite grain size during deformation.

**Figure 4:**
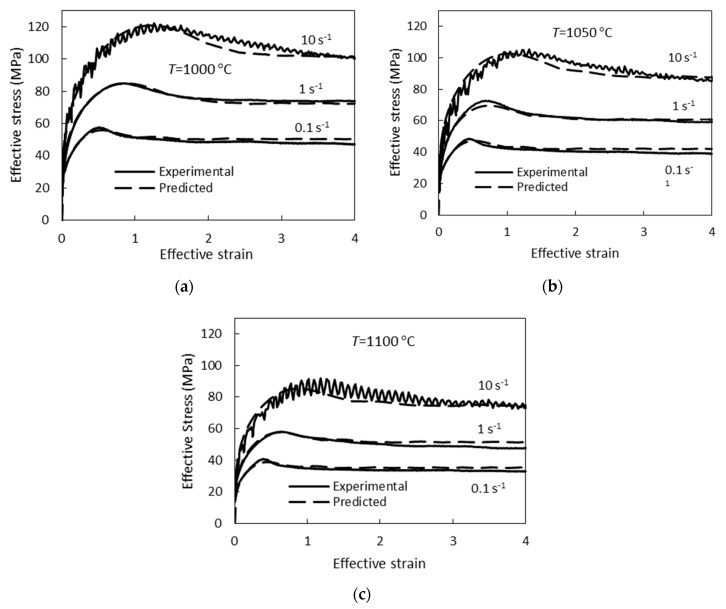
Comparison between the experimental and predicted flow curves determined at (**a**) 1000 °C, (**b**) 1050 °C, and (**c**) 1100 °C.

The developed mathematical model was validated by comparing the experimental curves of hot torsion and critical strains for dynamic recrystallization determined from these curves with the results of the calculation by the model. Experimental flow curves are compared with curves predicted by the developed model in Figure 4. Good agreement was obtained between the experimental results and the calculation—the greatest deviation of the experimental flow curves from the predicted curves is observed at a strain rate of 10 s^−1^, where the difference between the experimental and predicted flow stress values reaches 6%.

**Figure 5:**
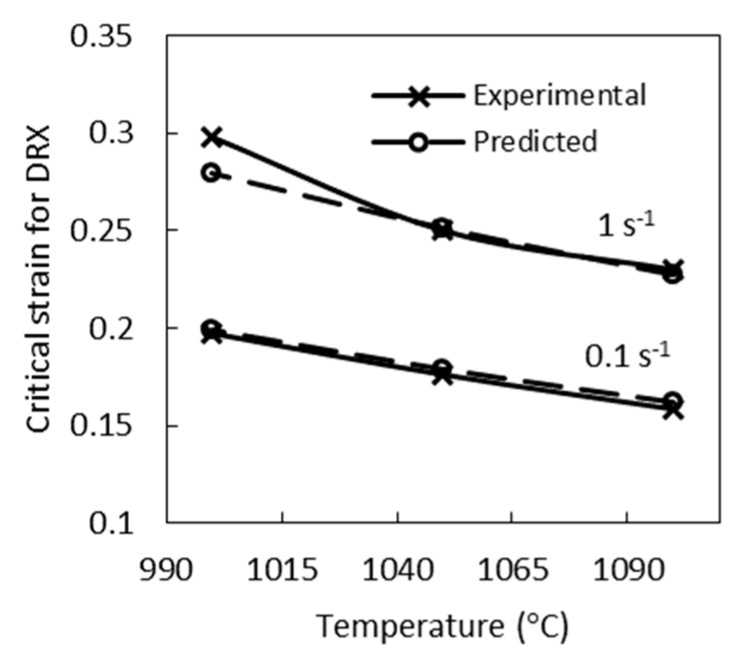
The dependence of the critical strain for dynamic recrystallization on the deformation temperature at a strain rate of 0.1 s^−1^ and 1 s^−1^.

The model study established that the experimentally determined values of critical strain for dynamic recrystallization are reached at the development of dynamic recrystallization by 2%. The results of predicting critical strain for dynamic recrystallization corresponding to the development of dynamic recrystallization by 2% are given together with the experimental values of $\varepsilon_{c}$ in Figure 5. The mean absolute error for predicting critical strain for dynamic recrystallization does not exceed 0.01.

**Figure 6:**
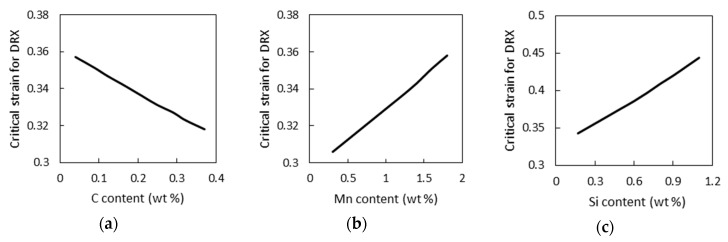
The influence of the content of (**a**) carbon, (**b**) manganese, and (**c**) silicon in low-alloyed steels on the critical strain for dynamic recrystallization.

The developed model was used to analyze the influence of the chemical element content in low-alloyed steels on critical strain for dynamic recrystallization. The 09G2 steel with 0.09% С; 1.6% Mn, 0.25% Si was used as the basic steel. The calculation $\varepsilon_{c}$ was conducted at successive variations of carbon, manganese, and silicon content in the limit range of their change in low-alloyed steels (0.04–0.37% С, 0.30–1.80% Mn, 0.17–1.10% Si). The austenite grain size before deformation and deformation parameters during calculation remained unchanged (*d*_0_ = 55 μm, $\dot{\varepsilon }$ = 1 s^−1^, *Т* = 1000 °C). The dependences of critical strain on the content of chemical elements in low-alloyed steels obtained in this way are given in Figure 6.

It is established that an increase in carbon content lowers critical strain for dynamic recrystallization while silicon and manganese increase this parameter. Changes in the chemical composition of low-alloyed steels of different grades can have a noticeable effect on critical strain. Therefore, at a deformation temperature 1000 °C and a strain rate 1 s^−1^, a change in the chemical composition in low-alloyed steels can lead to variations of critical strain from 0.271 to 0.462 (1.7 times).

In addition, the calculations showed that even with fluctuations in the content of elements within the steel grade composition, a noticeable change in $\varepsilon_{с}$ is observed. For instance, in the 09G2 steel, with the same deformation parameters, fluctuations in the content of elements within the grade (0.04–0.12% С, 1.40–1.80% Mn, 0.17–0.37% Si) can lead to a change in $\varepsilon_{с}$ from 0.332 to 0.377 (by 12.7%).

## 5. Conclusions

A mathematical model has been developed for predicting dynamic recrystallization and flow stress during hot deformation of carbon and low-alloyed steels, which takes into account the content of chemical elements in steel along with the deformation parameters. The model is based on the method of predicting the kinetics of dynamic recrystallization of steels with account of the information on the kinetics of their static recrystallization; the method can be used to calculate the recrystallization and flow stress of steels in the design and optimization of technologies associated with processes of hot deformation of metal, including hot rolling.
